# Representations of older people in Irish public broadcaster's online news coverage during the early stages of the coronavirus pandemic

**DOI:** 10.1177/07916035231183664

**Published:** 2023-06-26

**Authors:** Stephen Beasley, Virpi Timonen

**Affiliations:** 8809 Trinity College Dublin, Dublin, Ireland; Faculty of Social Sciences, University of Helsinki, Helsinki, Finland

**Keywords:** Coronavirus disease 2019, coronavirus, pandemic, older people, media, ageism, social constructivism, thematic analysis, Ireland

## Abstract

The media influences how we perceive and understand the world, groups of people, and events. The media has been criticised for portraying older people and aging predominantly negatively. In 2020, older people became the focal point of governmental responses in the management of the coronavirus pandemic. In this article, we explore how older people were represented in the officially most legitimate part of Irish news media during the first year of coronavirus disease 2019 pandemic. A sample of 137 articles was drawn from Ireland's national broadcasting company (Raidió Teilifís Éireann) online news content, and thematic analysis was applied to interrogate representations of older people in these data from a social constructivist perspective. Older people featured as ‘the face of COVID-19’ due to their constant and accentuated framing as especially vulnerable to the virus. Older adults were construed as a burden to the public, families, and the healthcare system and as a risk to healthcare workers. They were portrayed as powerless and rendered almost completely ‘voiceless’ as their own views rarely featured in the news content. The social constructions of older persons during the coronavirus pandemic reflected and exceeded the (typically implicit) ageism in contemporary western societies.

## Introduction

The media is a key source of information (Weimann, 2000) and the framing of this information influences our understanding of the world (De Vreese, 2005; Entman, 1993). The media can also contribute to greater social control of relatively powerless and disadvantaged groups (Tierney et al., 2006). In 2020 and 2021, the coronavirus pandemic featured prominent content in virtually all types of media (Farooq et al., 2020). Some aspects of media coverage of the pandemic (misinformation, framing stories in certain ways, and blocking or exaggerating viewpoints) have drawn criticism but the media has also been praised for playing an informative and educative role during the pandemic (Mutua and Ong’ong’a, 2020).

Public discourses concerning aging and older people have always been present in the media (Fealy et al., 2011; Miller et al., 2012, 2017), including Irish media (Fealy et al., 2011; Phelan, 2011). Positive aging stereotypes in the media include representations of older people as healthy and active, productive, wise, and as good grandparents (Allen and Wiles, 2014; Kessler et al., 2004; Rozanova, 2010; Whitfield, 2001). However, more commonly the media has been criticised for negative portrayals of older people (Horton et al., 2007; Loos and Ivan, 2018) as frail and dependent (Miller et al., 2017) and, during the coronavirus pandemic, as homogenous and vulnerable (Jen et al., 2021; Previtali et al., 2020). Such stereotypes obliterate older people's autonomy and ability to manage risks related to their own health and well-being (Morgan et al., 2021a).

In contrast to older ages typically being underrepresented in most media (Roy and Harwood, 1997), pandemic-era media coverage presents an opportunity to explore representations of older people at a time when the older population became a major focus for public health measures and media attention. Of course, media coverage of older people during the coronavirus pandemic was partly motivated by the higher risk of getting severely ill or dying from contracting coronavirus disease 2019 (COVID-19) among older cohorts (Ng et al., 2021; Sominsky et al., 2020; Tesch-Römer and Lamura, 2021) but this angle by itself would be a simplistic and one-dimensional way of approaching the issue. The use of stark cut-off points based on chronological age in public policy has been challenged (e.g. Häkämies-Blomqvist et al., 2002), yet the practice of age-based policymaking made a striking comeback during the pandemic when public health advice and directives were based on rigid age demarcations. While the international literature concerning older people during the pandemic has burgeoned (e.g. Ayalon, 2020; Chasteen et al., 2021; Reynolds, 2020), and there is some literature on the health and health care aspects of the older Irish population during the pandemic (Kennelly et al., 2020), social aspects such as media portrayal of older adults during the pandemic deserve more attention (Carney et al., 2022) and enable critical interrogation of how older adults are framed. This is important because representations and framings are not isolated from the lived experience of being an older person; this was particularly strikingly illustrated during the pandemic as older adults often structured their daily lives in accordance with what was advised or suggested in the media. While the literature on ageism is a useful starting point in broaching this topic (e.g. Palmore, 1990; Townsend, 1981; Walker, 2012), we argue that the treatment and social constructions of older persons during the coronavirus pandemic both reflected and exceeded the usually more implicit forms that ageism in contemporary societies takes.

The aim of this article is to explore how older people were represented in the most official and authoritative (public broadcasting) Irish online news media during the COVID-19 pandemic. Ireland's 2016 census recorded 637, 567 people aged 65 and over, and this number is estimated to more than double to 1.4 million by 2046. The number of people aged 80 and over is estimated to quadruple, reaching 484,000 by 2046 (CSO, 2017). Ireland is a relative ‘latecomer’ to population aging and research on intergenerational relations in Ireland evinces strong norms of solidarity towards older adults (Timonen et al., 2013); these features make Ireland a particularly interesting case for examining the social constructions of older adults at a time of crisis.

## Research approach

Social constructionism proposes that we make sense of the world through our social interactions with others by creating meanings and learning social norms that we perpetuate (Sudbery and Whittaker, 2019). For instance, retirement is taken for granted in many wealthy countries, yet retirement was instituted – socially constructed – at a particular time in history and in particular contexts, in response to the needs of capitalist modes of production, facilitating the distribution of work from older to younger people (Phillipson, 1982). Many everyday practices, including both written and verbal language, are also replete with social constructions of aging. Language is used to socially construct knowledge and understanding (Burr and Dick, 2017) including specific versions of events and portrayals of groups of people (Burr, 2015).

News media seeks to encapsulate (socially construct) events which in turn can influence public opinions about societal and political matters such as health and social care (Whittaker, 2012). In this research, analysing public service broadcaster news coverage of the pandemic provides insight into ways in which events and groups of people were portrayed in an ‘official’ news media. Analysis of media texts can take advantage of the relative ease of access to rich sources of data allowing for an efficient method of data collection (Whittaker, 2012).

### Data collection and analysis

Raidió Teilifís Éireann (RTÉ) is Ireland's public service broadcaster. RTÉ News is the number one news app in Ireland and RTÉ.ie is the number one multi-media website in Ireland (RTÉ Media Sales, 2020). Accordingly, RTÉ News online was chosen for its popularity and reach nationally, providing one data-rich source. Further, due to its nature as a public service broadcaster, the content from RTÉ News purports to represent the ‘official’ or ‘national’ perspective which at least in theory seeks to be informative and objective. Hence, we selected this media as representing the most officially legitimated source of news information available to the Irish public during the pandemic; as such, this source presents itself as ostensibly standing in contrast and opposition to misinformation, fake news and other discredited sources of information. The online content on the RTÉ website (and app) are in line with the other content (television and radio news) that the public broadcaster produces. Hence, while online news access might be limited among older adults, the articles contained in our data have a high degree of similarity with what is available from the more traditional media of radio and television news.

The sample of online news articles was drawn using the LexisNexis News and Business database. Two key search terms formed the basis for the search process: Older people and the pandemic. To improve the accuracy of the search results, variations were included for each search, for example the terms “old(er) people” – “older age” – “elderly” – “old(er) adults” were all included. For the pandemic, the search terms “pandemic”, “COVID-19” and “coronavirus” were all included.

English language articles only were included and a timeline set to help refine the quantity of articles returned in the search results. Due to the profusion of content, the search was limited to the first year of the pandemic and within this, two notable dates in the context of the pandemic were used to define the timeline; starting with the then Taoiseach, Leo Varadkar's public address on Saint Patrick's Day 17 March 2020 and ending on 20 October 2020, the beginning of the second round of Level 5 (most extensive) lockdown restrictions nationwide.

The search parameters yielded 190 articles from the LexisNexis database. Each article was screened for relevance to the research and purposive sampling was used to help judge which articles should be excluded. In total, 53 articles were excluded upon review, deemed irrelevant to the research, or consisting of international stories that did not impinge upon the Irish context of the research. The final sample size for data analysis consisted of 137 RTÉ news articles ranging from 200 words to over 6000 words.

Thematic analysis is a theoretically flexible method that identifies themes or patterns within qualitative data (Braun and Clarke, 2006). It allows for ‘thematising meanings’ (Holloway and Todres, 2003: 347), a suitable approach for establishing the main representations of older people in news media. Braun and Clarke (2006) characterise thematic analysis as ‘a flexible and useful research tool, which can … provide a rich and detailed, yet complex, account of data’ (p. 78). We adopted and applied Braun and Clarke's (2006) 6-step guide to maintain the advantages of the flexibility of thematic analysis whilst ensuring that it was carried out in a methodologically rigorous manner. Our analytic approach was data-driven (inductive) and encompassed the entire dataset. The first author read and coded the entire dataset, and the second author coded a selection of articles at the first stage of data analysis in order to establish agreement over coding practices. The selected articles were first coded using codes such as ‘COVID as a threat’; ‘protecting the vulnerable’; and ‘charitable acts towards older person’. The codes were then sorted into incipient, potential themes and following recursive rounds of analysis, codes were grouped into themes that represented the ‘best fit’ for each code. The themes were reviewed by the second author and both authors ensured that they were representative of the data within them and, in aggregate, of the dataset as a whole.

### Limitations

Limitations of the research include the timeline which runs from March until October 2020. Whilst this encompasses a significant period of time and yielded plentiful data, it does not capture subsequent developments regarding the representation of older people. This is pertinent considering the rapid changes in daily life during the pandemic and the long duration of restrictions in Ireland. Analysing media portrayals of older people during a longer period of time would allow insights into how these portrayals evolved (or remained similar).

Another limitation relates to the single news media source used which limits the generalisability of the findings (Morgan et al., 2021b). Other news media sources were not included, and the research does not include data from broader media sources such as social media or broadcast media. Comparisons of media types and outlets would allow insight into possible differences between them in portrayals of aging and older people.

Finally, notwithstanding the scope of the study, a mixed methods approach may have allowed for more comprehensive findings and an enhanced understanding of the research topic (Chow et al., 2010). For example, quantitative content analysis techniques could be applied to a larger sample that comprises both public service news services and other news outlets.

## Findings

Of the initial sample of 190 articles published between the above dates in March and October 2020, 137 met the inclusion criteria and constituted the data relevant to the purpose of this research. Analysis of these data yielded four themes and associated sub-themes:

1. Older people as the face of COVID-19 (sub-themes: The nursing home crisis; older people objectified by the virus; intergenerational division).
2. Older people as vulnerable (sub-themes: ‘at risk’; dependency and caremongering).
3. Older people as a burden (sub-themes: A burden of care; a burden for the public; a burden for families).
4. Older people as powerless (sub-themes: Powerless in the face of restrictions; powerless in decision-making; without a voice).

With some overlaps, 75 articles were thematically connected to the representation of older people as the face of COVID-19. In total, 82 articles were connected to the representation of older people as vulnerable. In total, 51 articles were connected to the representation of older people as a burden. In toal, 50 articles were connected to the representation of older people as powerless. The following sections outline these themes and the sub-themes within each.

### Older people as the face of COVID-19

This theme emerged from articles that positioned older people as central to the discussion around COVID-19, both directly and indirectly. This central positioning of older people was related to the three sub-themes discussed below. We start with the sub-theme that was the first to emerge in news reporting.

#### The nursing home crisis

The sharp rise in deaths in nursing homes internationally in the early days of the pandemic triggered acute media interest (Allen and Ayalon, 2021). Disturbing reports and images, as well as limited knowledge and understanding of the virus intensified the association of the disease with older people.

The data evidences a strong narrative of nursing homes as breeding grounds for the virus and featuring many deaths. A story titled ‘Donnelly (Minister for Health) claims 35% of staff in nursing home have virus*’* depicts a chaotic situation unfolding in nursing homes (ART19). The article revolved around the lack of governmental support, shortage of personal protective equipment (PPE) and staff and residents testing positive for the virus, mounting deaths, and under-resourced staff. This narrative continued, fuelled by headlines such as ‘335 outbreaks in residential centres “a huge challenge”’ (ART34) and stories headlining with deaths in nursing homes (ART36, 41).

As 2020 progressed, nursing homes became the focal point of debate as to how the response to the pandemic was handled. A story that reviewed what happened in nursing homes in the first few months of the pandemic was amongst the longest in the sample. Written through the lens of the nursing home crisis it also correlated with growing concern and fear among the public – ‘the first case of community transmission had been confirmed; someone had the disease in Cork, but no one knew how they got it’ (ART70)*.* Thus, the early months of the pandemic featured a profusion of articles evincing uncertainty, alarm, and frequent mention of severe disease and deaths in the older, institutionalised population in particular.

#### Older people objectified by the virus

The association between COVID-19 and older people manifested itself in content that came close to equating older people with the virus. This could be observed in the use of language and with particular reference to the effects of cocooning measures. The *‘*cocooning*’ of older people”* (ART3) and older people becoming ‘cocooners’ featured in multiple headlines (e.g. ART42 and ART78). A ‘COVID-19 dictionary for kids and adults who need it’ explains that ‘Nowadays cocooning means forming a protective barrier around older people, or those who have certain medical conditions, by reducing their close contact with other people’ (ART26). Homogenising and othering language was evident in articles covering provision of support services in which older people were referred to as ‘the elderly’ (ART42) or ‘the cocooned elderly’ (ART44). It is noteworthy that the term elderly was used in this media repeatedly despite its links to ageism and long-standing criticism of the media for its persistent use (McCárthaigh, 2020).

Descriptions of the ‘battle’ that healthcare workers faced in nursing homes and hospitals were closely linked to older people. The commonly used phrase ‘frontline staff’ featured alongside military and industrial terms such as ‘the coalface*’* (ART10), and dramatic statements and headlines such as ‘they have seen nothing like the threat coronavirus poses*’* (ART23) and ‘Virus kills two hospital workers*’* (ART31). Older adults with COVID were the frontline, the coalface, the threat that health care workers faced. Older people featured as spreaders of disease in the coverage of health service responses: ‘Patients discharged to nursing homes without test – NHI*’* (ART63 headline) began a series of articles that unveiled practices whereby older people were discharged from hospitals into nursing homes and transferred between facilities without being tested (ART70, 81, 94).

Older people were also framed as silent carriers of the disease, with ‘atypical’ or stealthy presentations of the virus that confounded and challenged professionals. A representative for the Health Service Executive (HSE) commented in one story; *‘*nursing homes are experiencing significant challenges because older people are presenting atypically for COVID-19*’* (ART34). In another story older people were statistically linked to the virus – ‘The latest Central Statistics Office [CSO] studies show that older people living in the most deprived areas have been the most adversely affected by COVID-19 and that this is strongly linked to outbreaks*’* (ART86)*.* In many cases older people were further objectified, anonymised, and defined by death and intensive care ‘numbers’.

Some stories attempted to decouple older people from the virus. The headline ‘WHO reiterates that coronavirus is a danger to children’ (ART18) is an attempt to counterbalance the (by then embedded) notion that COVID-19 is an older person's disease. Indeed, the association between age and the virus (perpetuated by the media) had become so strong that at times the media had to explicitly seek to combat it – ‘The very notion that ‘COVID-19 only affects older people’ is factually wrong’*.* This decoupling somewhat strengthened as the months progressed and more was learned about COVID-19. ‘The coronavirus lottery – It could be you*’* (ART109) sent the message that anyone can contract the virus. Clarifying words to this effect also came from the chair of National Public Health Emergency Team (NPHET) Irish Epidemiological Modelling Advisory Group – ‘In the early stages of the pandemic … the disease occurred across all age groups but was “particularly severe” for older people’ (ART111).

#### Intergenerational division

Portrayal of intergenerational differences increased in frequency as the months wore on. The Minister for Health reported ‘a third of new cases in recent days have been among people aged 35 or younger’ (ART83). Headlines such as ‘Young people driving some COVID-19 spikes – WHO’ (ART99) and ‘Pandemic now driven by 20s, 30s, 40s age group – WHO’ (ART106) appeared. This pattern intensified as ‘case numbers’ shifted from nursing homes to workplaces and communities at large (ART101, 108, 109, 111). This, in turn, reinforced the narrative of danger to community-dwelling older persons: when the Minister for Health was asked when it would be possible to hug grandchildren again, he said; ‘the reason we can't say you can safely hug your grandchildren is because that act of love could actually cause somebody to get very sick’ (ART59). The message is that all older people are extremely vulnerable to the virus, and that intergenerational contacts are dangerous and irresponsible.

Intergenerational conflict is featured implicitly in statements such as ‘The latest figures suggest that perhaps the intergenerational solidarity is cracking in some places’ (ART91). Extreme caution was advised in all contact with older people – ‘Everyone must be cautious in their interactions with these groups’; *‘*Those aged under 45 have a one in 100 chance of being hospitalised with COVID-19 but if you are aged 75, this rises to one in five’ (ART112). The irresponsibility of youth became a common trope during the summer months – ‘Images of reckless partying have also contributed to concerns that the virus could leak into the community and put vulnerable people in particular at risk’ (ART106) – and stood in contrast to portrayals of ‘compliant’ older people: ‘People over 70 have been among the most compliant during the pandemic … the new government advice for people over 70 may taste like a bitter pill for some’ (ART106). Such portrayals of intergenerational division reinforced the narrative of older people as the face of COVID-19.

### Older people as vulnerable

#### **‘**At risk’

Throughout the data older people were consistently grouped with those ‘medically at risk’ of COVID-19. There was no discussion as to what constituted being ‘medically at risk’ yet it was connected to old age in particular. A sense of normality emerged in the reporting of negative outcomes from COVID-19 in conjunction with age, illustrated by statements such as ‘The deceased were aged between 66 and 84’ (ART31). An early story in the sample provided insight by focusing on a retired General Practitioner who, at 75 years of age, ‘is himself in the at-risk category for COVID-19. He also has a heart condition, which makes him even more vulnerable’ (ART10)*.* Shortly after cocooning was introduced, an article titled ‘What you need to know if you are cocooning’ issued drastic, blanket instruction in an authoritative tone – ‘people aged over 70 and people who are extremely medically vulnerable are to remain at home and not venture out at all but instead “cocoon” themselves from COVID-19’ (ART17). An article that defined vulnerable groups as ‘people who are more likely to get the virus, such as the elderly or people who already have certain medical problems’ (ART26) indicates confusion between the risk of severe illness and the risk of contracting the virus and is an example of (inadvertent) misinformation.

Associating loneliness, isolation and fear with older people contributed to the vulnerability narrative. Around the time when cocooning advice was first issued, one story predicted accurately that ‘cocooning measures will also result in lots of older people feeling lonely and isolated’ (ART17). Later in the sample, in October 2020, an older woman is reported to be lonely and fearful since her husband died. She stated that ‘cocooning has made old people afraid’ (ART125). Another story titled ‘Without them I had no one – loneliness in a pandemic’ detailed non-governmental organization (NGO) services for older people experiencing loneliness and social isolation (ART66) which had – unsurprisingly – increased as a result of the cocooning and similar advice issued to older persons.

#### Dependency and caremongering

Dependency is closely associated with vulnerability, as isolated (‘at risk’, ‘cocooning’) individuals evidently require the help of others and are likely to become dependent on others due to their inability to shop, for instance. Dependency is emphasised early in the sample with an article titled ‘Points of contact key for elderly during COVID-19 crisis’ with a paternalistic tone asking us to ‘spare a thought for older people in these extraordinary times’ (ART4)*.* Other articles in the sample illustrate older people's dependence on home care services (ART32) and community services (ART77).

Examples of ‘caremongering’ were identified in 26 articles that illustrate caremongering in care settings and in the community. Caremongering has been defined as behaviours and statements by (typically) younger, able-bodied individuals (often on social media) that purport to help people who are (or are perceived to be) at greatest risk of severe COVID-19 infection (Vervaecke and Meisner, 2021). One story detailed an appeal issued on social media by healthcare staff in a nursing home to help ‘lift the spirits’ of residents who were ‘no longer allowed to receive visitors due to social distancing measures brought in to protect the vulnerable and elderly’ (ART13)*.* The post went viral on social media with the nursing home receiving messages and letters from around Ireland and Europe. Another story outlined a similar experience of staff in a hospice raising funds through public appeal to provide iPads for patients to keep connected (ART33). Any verbatim expressions of older people were notably not included in these two stories despite the article emphasising strong emotions (such as joy) among them.

Many of the articles provided examples of caremongering that championed the contribution of people performing acts of kindness. It was also clear from the stories that caremongering is not entirely selfless as the sense of fulfillment and purpose on the part of the contributors was frequently emphasised. The benefits of caremongering were visible in the stories but it was also clear that the blanket introduction of cocooning measures had created and exacerbated dependency for older people. At the same time roles were created for others to meet those needs through caremongering, enforcing the narrative that older people are vulnerable and helpless. This also fits within a wider context of exhortations to protect the under-resourced healthcare system (in particular, the limited number of hospital beds) through individual actions.

Some signs of resistance to the dependency created as a result of cocooning emerged towards the later articles included in the sample in autumn 2020 when the prospect of new restrictions was looming. A story titled ‘Older people resolute in maintaining independence’ (ART135) goes on to state ‘While many will adhere to the Government advice, others are more resolute in maintaining some form of independence, given their experience earlier in the year’. The story included views from older people with one woman's experience of the debilitating effects of restrictions imposed upon her evident in the words – ‘I'm not doing it again. They can jail me, I don't care. I'm getting my own groceries’*.* However, such ‘declarations of independence’ were rarely featured and represented the few occasions on which older persons were directly quoted in their own, critical words.

There were only three articles in the sample that contained explicit challenges to the vulnerability narrative around older people. These featured a consultant geriatrician raising concerns about the stereotypical view of old age being compounded by cocooning (ART43); the WHO reiterating that ‘age is not the only risk for severe disease’ and that those in every age group can be vulnerable (ART18); and an NGO for older people highlighting that with the introduction of cocooning came ‘the constant messages that you’re vulnerable’ (ART125).

### Older people as a burden

#### A burden of care

The burden of care has some ‘objective’ foundations in the fact that those in older age are more at risk of severe illness from COVID-19 and are more likely to be hospitalised (Ng et al., 2021; Sominsky et al., 2020; Tesch-Römer and Lamura, 2021). The first article in the sample ‘Nursing homes launch recruitment drive due to COVID-19’ drew attention immediately to the impact of the virus on nursing homes and the care needs of residents. The high care needs of older people were underlined in one story which tells us that almost 11,000 Special Needs Assistants (who normally work in schools that had been closed due to pandemic restrictions) had responded to reassignment measures ‘introduced to help deal with the consequences of the COVID-19 pandemic’.

The spread of virus infections and resident deaths in nursing homes, shortage of care staff and PPE and care staff contracting the virus were just some of the features of early discussion (ART10, 19, 23, 28 and 32) that contributed to depicting older people as a burden and risk to healthcare staff. Nursing homes and acute settings were ‘the frontline’ – ‘Every worker in the health service is on the frontline and they all need PPE to be able to do their job’ (ART19) where older people were (implicitly) portrayed as a risk to those that come into contact with them – ‘Each day at work, nursing staff and other health care workers may face a clear and present danger of contracting the virus’ (ART48).

Pressures continued to be identified around ICUs (ART52), the need for the HSE to use private hospitals to provide care and clear waiting lists (ART57), the impact on non-COVID-related care (ART61, 86), and reminders of pre-existing pressures on system particularly during the winter (ART91, 120), the general tone illustrated by ‘The health system is noticeably under strain now, with increased admissions to hospitals and intensive care units (ICUs), and some nursing homes under significant pressure’ (ART27).

Praise for healthcare staff contributed to strengthening the narrative of older people as a burden – ‘We hail the health staff working in unprecedented conditions. They have always cared for people with serious illness and facing death as part of their daily work, but yet they have seen nothing like the threat coronavirus poses’ (ART23). Healthcare professionals, particularly in nursing homes and acute settings, were consistently portrayed as risking their lives in the face of the virus. An ambivalent dichotomy of care and blame thus emerged as the ‘vulnerable’ older people are also portrayed as the main source of the risks healthcare staff are taking, and by extension as a burden on the healthcare system.

As the narrative started to change regarding risk to younger age cohorts, the theme of older people as a burden on the health system seemed to be further substantiated – ‘Health officials take the view that the current lower number of patients being admitted to hospital is likely due to the younger profile of cases being seen now’ (ART118). Similar pressures could also be observed in relation to home care and the high unmet need for home care amongst older people was illustrated by extensive waiting lists (ART37, 127). The connection to pressures in acute settings is evident in an advocacy agency representative's statement that ‘some people waiting up to a year in acute hospitals for home care were suddenly allowed to go home with supports to free up beds for the COVID-19 crisis’ (ART37). Depictions of older people and the health and social care system positioned them as intricately connected. A representative of the HSE is quoted saying the organisation had ‘never seen so much change implemented in such a short time’ and this ‘puts us in good stead to look at issues in the future, including how we care for the elderly and the private/public hospital system’ (ART57). By extension access to the system for the rest of the public is also dependent upon this relationship between older people and the system – ‘when the virus is under control, the response capacity of the public health system will be the key thing to protect us’ (ART136). The question remains as to whether the extreme cocooning measures were introduced to protect older people or the (comparative limited) capacity of the acute health care system.

#### A burden for the public

Early in the selected coverage, the Minister for Health advised on cocooning whilst praising the enormous efforts required by healthcare professionals, emphasising the importance of adhering to public health advice to support healthcare professionals across the system (ART3). The relationship between older people and the public continued to be illustrated throughout with the NPHET stressing that ‘there is simply no way of protecting nursing homes or any other institutional setting if we don't control the spread of this infection in the community’ (ART65)*.* Hence, the messaging was clear that everybody had to be involved in the collective task of protecting ‘the vulnerable’ older persons.

‘What you need to know if you are cocooning’ covered the support measures put in place across society to enable those over 70 to stay at home (ART17), opening up debate as to whether those measures were helpful or oppressive as they created an environment where older people did not have any excuse not to abide by the rules. This was exemplified in an article reporting ‘concerns among pharmacists and the Irish Pharmacy Union that not everyone over 70 is adhering to the cocooning measures’, elaborating that ‘While it can be difficult for those who are fit and well and who enjoy getting out and about to remain inside, it's in their safety interests and that of others to do their best to adhere to the guidance. Others should be able to assist in getting prescriptions filled, collected and delivered for people who need to stay at home’ (ART23). Article 43 quoted a consultant geriatrician saying, ‘Many over 70s report being looked at disapprovingly if they are outside their home’. In other words, at times it even became socially unacceptable for older persons to exhibit (safe) behaviours such as going for a walk outside; the negative reaction to such behaviours illustrates the perception of older persons as a collective burden of care and concern.

Older people were portrayed as a barrier to effective rollout of the COVID Tracker App due to many people over 65 not owning a smartphone (ART74). A story on the progress of vaccine candidates urged caution that more research was needed, ‘particularly among older adults who are disproportionately at risk of dying of COVID-19’ (ART96)*.* In ‘Have we passed the 5 tests to lift virus restrictions?’ older people were not specifically mentioned but easing of restrictions was said to be dependent on factors such as the number of people in ICUs and ‘resilience of our hospitals’ and the ‘ability to shield and care for at risk groups’ (ART56). A picture of older people holding back the fight against the virus emerged from such coverage. This in turn created a sense of wider society having to suffer and sacrifice because of those who are worst affected by the virus.

The burden for the public was portrayed also through the changes that businesses such as those in retail needed to make to protect public health (ART58, 64). The fear and guilt of potentially causing infection for older persons was evident from this statement by the owner of a business who had decided to stop operating: ‘I have staff with elderly parents and if they ended up bringing this disease home, I would be waking up in the middle of the night saying it was my fault’ (ART58).

#### A burden for families

Older people were portrayed as a burden for family members and loved ones, evidenced by statements such as ‘Their (older persons’) mental health can suffer, with having to stay at home all the time and it also adds to pressure on families, who care deeply for loved ones’ (ART91), and ‘For over 1000 people here, the virus has resulted in death and devastation for their families and friends left behind. The healing will be long and difficult’ (ART48). Concerned family members abroad asked for assistance from community groups for older family members at home (ART68) whilst another story detailed concerns families had with restrictions imposed on nursing home residents and cessation of visits (ART70).

However, the circumstances surrounding nursing home deaths constituted the most visceral form of burden – ‘Of course, families are dealing with extraordinary challenges too. It is almost unimaginable to think that people who are dying with a COVID-19 related illness cannot be visited by relatives’ (ART23). The burden resonated in ‘COVID-19: the loss of a loved one’ (ART85) which highlights the work of healthcare professionals in ensuring those dying from COVID-19 were as comfortable as possible in their last moments. The pain associated with not being able to be by the side of an older family member when they die was highlighted and extended elsewhere to the pain associated with restrictions preventing attendance at funerals (ART115). Feelings of anger, pain and regret for family members who lost loved ones in nursing homes were also described (while implying that this was an unavoidable consequence of protective public health measures). A nursing home manager commented: ‘It's been the most stressful time that I've had in the last 24 years. And it was kind of … emotional because we know the people so well. So to deal with all the families, it was hugely, hugely difficult’ (ART94).

### Older people as powerless

#### Powerless in the face of restrictions

Although restrictions in response to COVID-19 were applied across all age groups, the analysis revealed that restrictions in the form of cocooning for older adults in the community and strict regimes for nursing home residents were the focus of related content. Early in the sample, an article outlines that nursing home residents ‘are no longer allowed to receive visitors due to social distancing measures brought in to protect the vulnerable and elderly’ (ART13)*.* Another story discussed the difficulty and impact of visitor restrictions – ‘It has been a difficult time for residents missing face-to-face contact with their loved ones (…) It is a decision made with great difficulty, but it has to be made for the safety of all and people have been very good about it’ (ART33). The phrase ‘very good about it’ indicates absolute and unquestioning quiescence – or resignation and lack of choice, both hallmarks of powerlessness.

It was noticeable how the language around restrictions changed or softened over time. At the beginning of the sample we are told ‘people aged over 70 and people who are extremely medically vulnerable are to remain at home and not venture out at all but instead cocoon’ (ART17)*.* In contrast to such firm directives, at a later stage people aged 70 and over – a category that was equated with the ‘medically vulnerable’ – were told they ‘should limit interactions and stay at home as much as possible’ (ART103). This was reinforced by the Irish President who is quoted saying ‘there were mistakes made in the language used in relation to older people … we should not have “fudged” what was mandatory and discretionary’ (ART71). The discretionary advice was interpreted by many older adults as mandatory (in no small part due to the tone of media coverage that was replete with exhortations to stay indoors), leading to unhealthy behaviours such as withdrawal from any forms of exercise (Bailey et al., 2021), later shown to be ill-informed and damaging as outdoor activities with social distancing are extremely low-risk and conducive to better overall health and wellbeing.

#### Powerless in decision-making

Powerlessness was evident in decisions relating to cocooning and nursing homes by committees that lacked any representation of older people. Some sense of unease with this emerged as 2020 progressed and questions were asked, particularly in relation to nursing homes: ‘Ministers urge Holohan (Chief Medical Officer) to ease restrictions on elderly’ (ART47), The Minister for Health was quoted saying that ‘he was conscious of how difficult it was for older people to cocoon … the Government would monitor how cocooning could evolve as appropriate’ (ART49). The extent and severity of cocooning indicate how older people's lives were influenced by strongly worded media messaging.

Approximately halfway through the sample of articles, intensive discussion appeared about hat happened in nursing homes. One story stated that ‘Nursing Homes Ireland will say that key State organisations left the nursing home sector and its residents “isolated” in the early days of COVID-19’ (ART63)*,* and another titled ‘Two months: How COVID-19 hit Ireland's nursing homes’ (ART70) summarised many of the decision-making issues. A picture is painted that the fate of residents was in the hands of others and that breakdowns in lines of communication between Nursing Homes Ireland, the Health Information and Quality Authority, Department of Health, the HSE, and the Minister for Health produced negative outcomes for some older persons who were left without any latitude of independent decision-making.

The lack of involvement of older people in decision-making was acknowledged in a very small number of articles. A consultant geriatrician in one instance suggested that older people ‘should be allowed to lead discussion on cocooning’ and have representation on the NPHET (ART43). In another, a representative of an NGO for older people commented that ‘older people are best placed to make decisions about what is right and important for them’ (ART125)*.* These were in a very small minority of articles featuring the idea that older people should have at least some choice regarding the level of risk they were prepared to live with. Absences and silences are significant in discourses that pertain to power: the idea that an older person might want to increase marginally their risk of exposure in return for some much-needed social interaction during their (in some cases very limited) remaining lifespan was an idea that did not feature at all in the sample of articles, indicating that this was officially ‘unthinkable’.

Towards the end of the sample, a journalist referenced a Canadian study hypothesising that the risks associated with visitor restrictions in nursing homes outweigh the potential benefits linked to preventing COVID-19 infections (ART129). The impact of severe restrictive measures on older people's health and well-being was raised early in the pandemic (e.g. Armitage and Nellums, 2020) but such evidence was superseded by the overwhelming medical and moral pressure to ‘do the right thing’ as determined by a short-term view of public health that left no room for an older person's autonomy or agency or consideration of their broader health and wellbeing.

#### Without a voice

Older people were seen to be without a voice in the sample through the effects of the pandemic but also the lack of older persons’ views expressed or sought after in the content of the articles themselves. The voicelessness of older people was evident in responses to the nursing home crisis where public concern and the media were the leading voices. Residents who died were anonymous, referred to as ‘the deceased’ (ART31)*,* only defined by their age. Sympathies were expressed towards friends and families of residents who died in stories that featured expressions such as an HSE representative stating ‘our thoughts are with them at this difficult time’ (ART37). Expressions of reassurance were directed at relatives and next of kin rather than older people living in nursing homes or in the community.

The analysis revealed that older people's voices were distinctly underrepresented. This underrepresentation is compounded by the content of the articles which is directly relevant to and impactful upon older age cohorts. Other voices spoke on behalf of older people, notably charities and NGOs in the age sector. Fewer than 20 articles in the sample included quotations directly from older people, many of which were minimal or paraphrased. Several articles had sought the experiences of a small number of older people cocooning with their views contributing to stories reflecting compliance (ART4, 110, 125), resilience and recovery from the virus (ART21, 50), loneliness in the pandemic (ART66) and gratitude for supports provided (ART42, 44). Two stories also included the views of retired healthcare workers, underlining responsibility and compliance regarding public health guidelines (ART8, 10).

Older people are frequently framed as inactive economically or no longer of any use to society. In our sample, this idea is not conveyed explicitly but rather ‘between the lines’. In one story covering cocooning measures, a TD was quoted saying his 94-year-old mother was ‘very happy as she is doing something for Ireland again’ (ART7). This relates to the widespread narrative of uselessness in older age, which the mother then disrupts, ‘becoming useful’ once again, by dutifully cocooning. The paradox of ‘becoming useful’ by doing nothing and isolating from society throws into sharp relief the taken-for-granted uselessness of older people.

The rare cases of ‘active’ older people that are featured in the sample provide a useful foil for interrogating the image of a ‘suitably productive’ older person. A story featuring extracts from an interview of a retired man, opened with ‘Meet the 75-year-old retired GP joining the fight against coronavirus. He's got a pacemaker, is fitted with a stent in his heart, and his family would prefer to lock him indoors’ but he ‘is coming out of retirement to help battle COVID-19’ (ART10). The underlying narrative of general uselessness in older age is accentuated through the praise for the ‘exceptional’ case. It is also noteworthy that the article highlights the ‘bravery’ of a *male medical professional* – another silence that speaks volumes is the absence of any stories of the older women who looked after grandchildren or worked in non-medical settings.

## Conclusions

This research examined representations of older people in Irish public service news media during the early stages of the pandemic. Older people featured as the face of COVID-19, closely associated with the virus in a variety of contexts, first in institutional care settings and subsequently as ‘cocooners’ in the community. The threat of the virus to older age groups was linked to portrayals of older people as a vulnerable group. Media coverage of caremongering – well-intentioned but often paternalistic acts of kindness towards older adults – contributed to this representation. Older people were also depicted as a burden to the healthcare system and as representing a risk to healthcare workers in nursing homes and acute settings. They were portrayed as a burden for the family and the rest of the public. Lastly, older people were construed as powerless. This powerlessness was notable in the form of strongly worded cocooning advice in the community and lengthy, extreme visitor restrictions in nursing homes. These framings of older people resulted from well-intentioned but paternalistic efforts to protect older adults’ physical health, with little concern for their broader well-being let alone autonomy. The analysis also found that older people's own voices, especially critical ones, were distinctly underrepresented in media coverage pertaining to COVID-19. Quotations from older people rarely featured and other voices – such as charities and NGOs in the age sector – spoke on behalf of older people.

The four themes are distinct but also interconnected. The representation of older adults as vulnerable brings forth protective responses, which in turn feed the representation of older people as a burden. However, there are also some apparent contradictions. The voicelessness of older people is surprising in view of fact that the media strongly contributed to older persons becoming ‘the face of COVID,’ especially during the early stages of the pandemic. The burden and vulnerability discourses help to make sense of the powerlessness: groups that are construed as burdensome and vulnerable cannot be heard due to their vulnerability and need for protection and they do not deserve to be heard as they are burdensome. Indeed, it is easy to see the continuity between the themes identified in our research and Townsend's enduring observation that dates to empirical research conducted decades earlier: ‘The elderly are usually viewed as the grateful and passive recipients of services administered by an enlightened public authority. This can but reinforce their dependency both in their own eyes and that of the public’ (Townsend, 1981). While the representations of older adults during the pandemic, therefore, chime with the extensive literature on structured dependency and ageism (Palmore, 1990; Phillipson, 1982; Walker, 2012), they are also manifestations of broader relationships of power in contemporary societies, further exacerbated by the speed and extent of online communications, including and perhaps especially various news media. Interrogating the role of political, medical and media actors in shaping the kinds of representations that we have outlined in this article is a topic that calls for further research.
